# Discovery of a chemical small molecule inducing umbilical cord mesenchymal stem cell differentiation to vascular endothelial cells

**DOI:** 10.1186/s13619-025-00278-2

**Published:** 2026-01-16

**Authors:** Bangzhao Zhou, Xiaohui Chi, Xinyu Xie, Baoxiang Zhao, Li Wang, Junying Miao, Zhaomin Lin

**Affiliations:** 1 Shandong Provincial Key Laboratory of Development and Regeneration, School of Life Science, Shandong University, Qingdao, 266237 People’s Republic of China; 2 Institute of Medical Science, the Second Qilu Hospital of Shandong University, Jinan, 250033 People’s Republic of China; 3 Institute of Organic Chemistry, School of Chemistry and Chemical Engineering, Shandong University, Jinan, 250100 People’s Republic of China; 4 State Key Laboratory of Ophthalmology, Guangdong Provincial Key Laboratory of Ophthalmology Visual Science, Zhongshan Ophthalmic Center, Sun Yat-Sen University, Guangzhou, 510060 People’s Republic of China

**Keywords:** Chemical small molecules, Human umbilical cord mesenchymal stem cells, Endothelial differentiation, Single-cell RNA sequencing

## Abstract

Human umbilical cord mesenchymal stem cells (hUC-MSCs) have emerged as promising candidates for clinical applications in vascular disease therapy and in the in vitro modeling of vascular regeneration. However, the translational potential of hUC-MSCs requires direct differentiation into functional vascular lineage cells, particularly vascular endothelial cells (VECs) and endothelial progenitor cells (EPCs). A critical challenge is the lack of reliable sources that yield sufficient quantities of mature VECs/EPCs for therapeutic purposes. To address this limitation, we established an efficient protocol for generating VECs from hUC-MSCs. Preconditioning hUC-MSCs using small molecules with cytoprotective properties can enhance their potential for use in cell-based therapeutics. Through systematic screening, we identified CPP as a novel small chemical molecule that effectively induces the endothelial differentiation of hUC-MSCs. Remarkably, our CPP-based induction protocol achieved > 90% conversion to functionally competent VECs within 5 days, as evidenced by both in vitro assays and in vivo functional validation. Single-cell RNA sequencing (scRNA-seq) analysis further delineated the differentiation trajectory and confirmed the acquisition of endothelial-specific molecular signatures during lineage commitment. These findings establish CPP as a potent inducer of rapid endothelial differentiation, and provide mechanistic insights into stem cell fate determination.

**Graphical Abstract:**

Take home figure CPP drives the differentiation of human umbilical cord mesenchymal stem cells (hUC-MSCs) into functionally competent vascular endothelial cells (VECs). Crucially, the long non-coding RNA MEG3 acts as a pivotal regulator within this differentiation pathway.

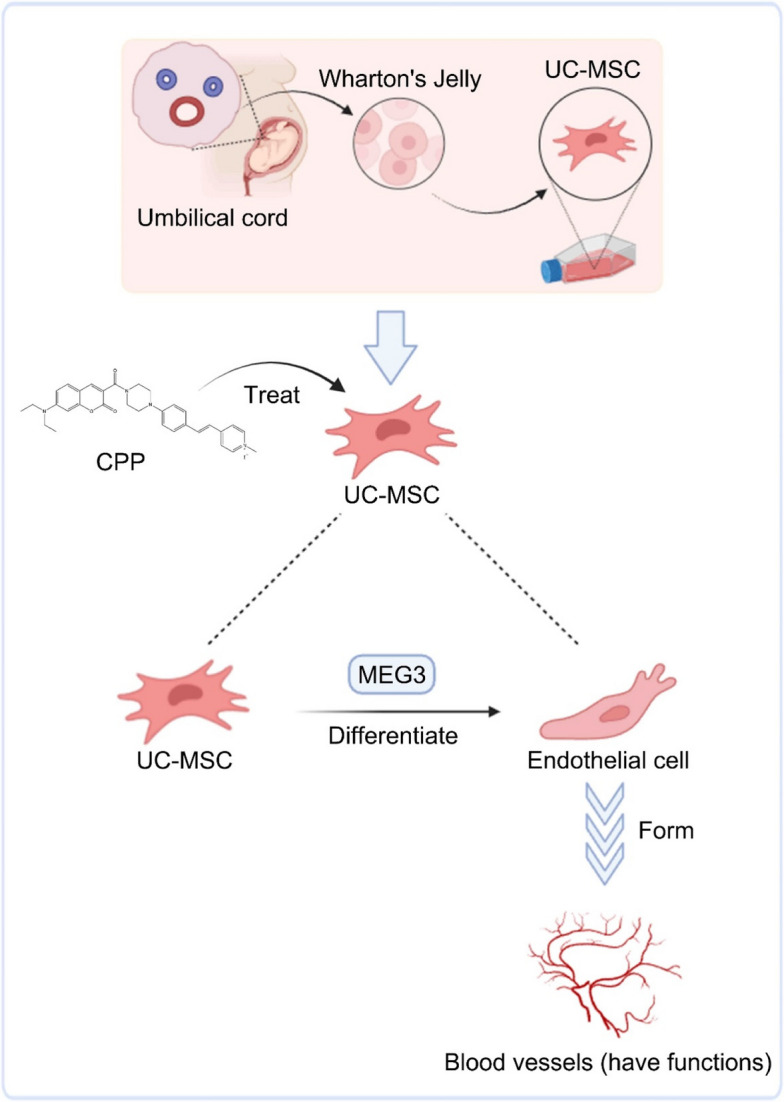

**Supplementary Information:**

The online version contains supplementary material available at 10.1186/s13619-025-00278-2.

## Background

Cardiovascular diseases (CVDs) are the primary cause of global mortality and were responsible for 32% of deaths worldwide in 2022 (Global Regional 2013; Woodruff et al. 2024; Choi et al. 2009). In particular, critical limb ischemia (CLI), the end-stage manifestation of peripheral artery disease, shows devastating outcomes; 43% of patients undergo major amputation within 5 years despite maximal medical therapy (Howard et al. 2015). Patients with diabetes face accelerated disease progression, exhibiting a 3.7-fold higher CLI incidence than nondiabetic cohorts (Peripheral arterial disease in people with diabetes 2003), with > 60% being ineligible for conventional revascularization due to diffuse microvascular pathology (Gupta and Losordo 2011). This vulnerability stems from hyperglycemia-induced microvascular dysfunction mediated by AGE-dependent endothelial dysfunction, pericyte loss, and neurovascular unit destabilization (Pacinella et al. 2022).

Cell-based revascularization offers promise for limb salvage (Tompkins et al. 2018), yet current strategies face significant limitations: (I) Progenitor cell approaches such as bone marrow-derived endothelial progenitor cells (EPCs) exhibit proangiogenic capacity but suffer from scarcity and isolation challenges (O’Neill 2005; Liu et al. 2013). (II) Pluripotent stem cell derivatives, such as hESC-ECs and iPSC-ECs face ethical constraints, technical complexity, prohibitive costs, and unresolved gene editing risks (Khan et al. 2018; Golchin et al. 2021; Liu and Zheng 2019). (III) Clinical evidence gaps; meta-analyses (16 randomized clinical trials, *n* = 774) showed reduced amputations versus untreated controls but lack placebo-controlled efficacy (Liew et al. 2016).

To address these barriers, human umbilical cord-derived MSCs (hUC-MSCs) present a compelling alternative with inherent advantages such as accessible sourcing (Zhang et al. 2023), minimal harvest morbidity (Mushahary et al. 2018), low immunogenicity (Li et al. 2015), multilineage potential (Xu et al. 2024), scalable expansion (Todtenhaupt et al. 2023), and established safety (Huang et al. 2024). However, precise control of hUC-MSC differentiation into functional vascular endothelial cells (VECs) remains a critical challenge.

Early methods for inducing endothelial differentiation from stem cells often involved co-culture with mouse bone marrow-derived stromal cell lines (e.g., OP9 or M10B2) in a non-directed manner (Choi et al. 2009). However, this approach resulted in low differentiation efficiency and heterogeneous cell populations, failing to meet the requirements of regenerative medicine. The EB method leverages the spontaneous differentiation of stem cells within three-dimensional aggregates to mimic early embryonic development, yet the process is poorly controllable, and endothelial cell yields remain limited (Adams et al. 2013; Bai et al. 2013). An alternative strategy involves culturing stem cells as a monolayer on matrix-coated plates and guiding their stepwise differentiation via the mesodermal stage through timed addition of specific factors (Sahara et al. 2014). Most protocols under this strategy consist of two main phases—mesoderm induction and endothelial induction—each relying on distinct growth factors and culture conditions.

Due to the absence of key in vivo cues such as blood flow, pulsatile pressure, and tissue-specific microenvironments in vitro, the resulting endothelial cells often display an ambiguous phenotype, being neither typically arterial nor venous. Studies have shown that endothelial cells derived via the EB method exhibit heterogeneous expression of arterial, venous, and lymphatic markers, though their differentiation toward specific lineages can be directed by modulating culture conditions (Rufaihah et al. 2013).

Through our research, we established a pharmacological solution. We leveraged small molecules to direct hUC-MSC differentiation via stress-responsive pathway activation (Qin et al. 2017; Xie et al. 2017). The core of this strategy is CPP, a novel synthetic coumarin derivative (4-(4-(7-(diethylamino)-2-oxo-2H-chromene-3-carbonyl)piperazin-1-yl)benzaldehyde (1) and 1,4-dimethylpyridin-1-ium iodide). Our earlier studies have demonstrated its ability to induce differentiation of human dermal fibroblasts into vascular endothelial cells, and further investigations revealed that CPP can effectively drive the differentiation of UC-MSCs into VECs (Zhang et al. 2018). Through single-cell RNA sequencing, we mechanistically dissected CPP-induced vasculogenesis and uncovered the long noncoding RNA MEG3 as the pivotal regulator governing the proangiogenic function of CPP (Li et al. 2022).

## Results

### CPP initiates phenotypic remodeling in human umbilical cord-derived MSCs

**Fig. 1:**
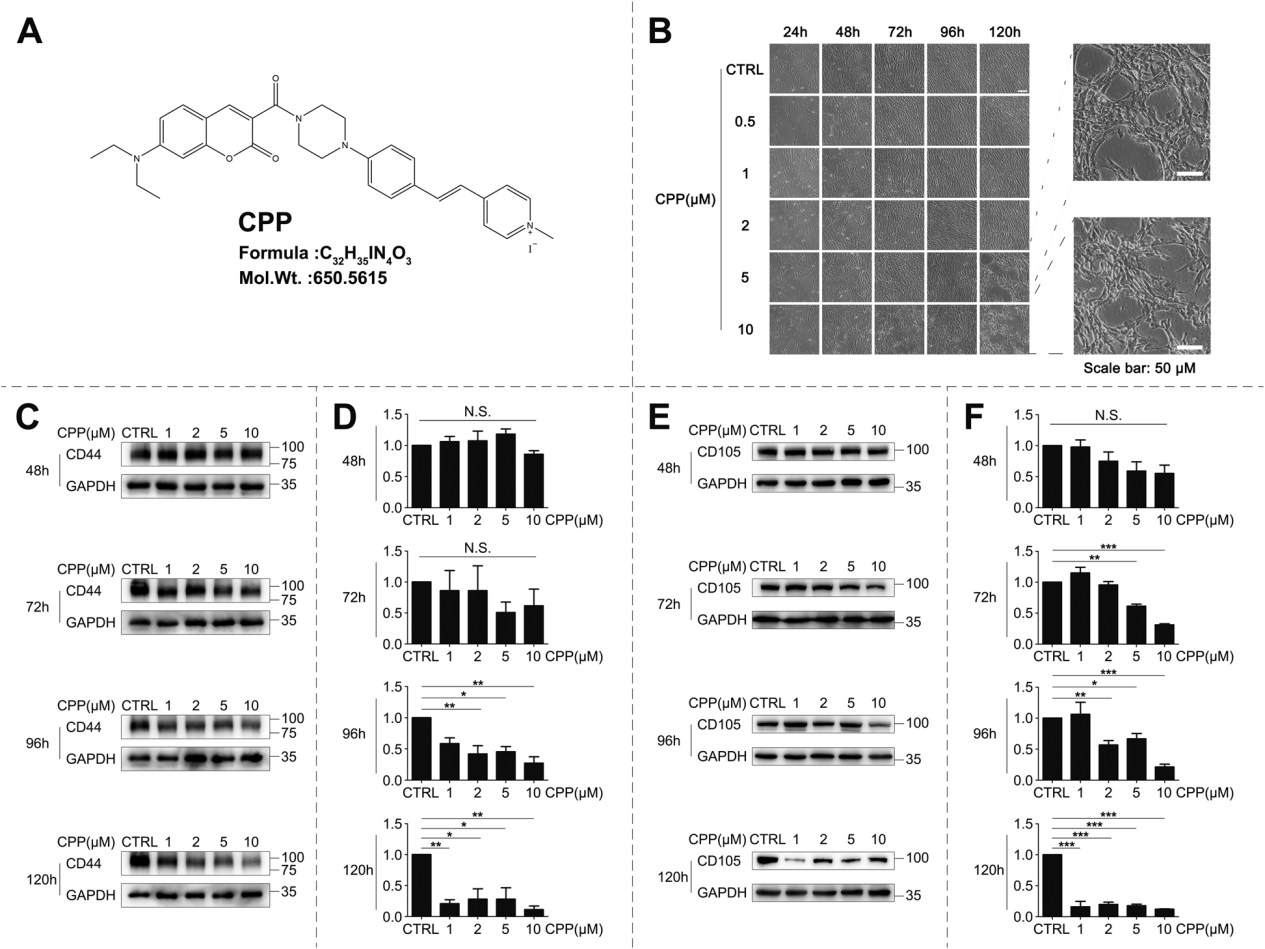
CPP Induces Phenotypic Remodeling of UC-MSCs. **A** Chemical structure of CPP. **B** hUC -MSCs were continuously induced by different concentrations of CPP (0, 0.5, 1, 2, 5, 10 μM) for 120 h and observed continuously using an inverted phase contrast microscope. Scale bar: 50 μm. **C** hUC-MSCs were treated with different concentrations of CPP (0, 1, 2, 5, 10 μM) for 48, 72, 96, and 120 h, and the changes in CD44 protein levels were detected by Western blot. **D** Quantification of CD44 Western blot bands using GAPDH as the loading control. **E** hUC-MSCs were treated with different concentrations of CPP (0, 1, 2, 5, 10 μM) for 48, 72, 96, and 120 h, and the changes in CD105 protein levels were detected by Western blot. **F** Quantification of CD105 Western blot bands using GAPDH as the loading control

Through systematic small molecule screening, we identified CPP, a coumarin derivative (Fig. 1A), as a candidate modulator of hUC-MSC phenotypic plasticity. To exclude potential drug toxicity, we first assessed the viability and quantity of hUC-MSCs using the CCK-8 assay. It is worth noting that CPP exposure showed no significant impact on either cell number or activity. (Fig. S1A), hence confirming its biocompatibility. CPP treatment induced profound morphological alterations in hUC-MSCs, marked by spontaneous lacunar structure formation and development of complex cellular networks in vitro (Fig. 1B). These architectural reorganizations implied a potential lineage transition. We validated this cellular differentiation molecularly by conducting quantitative analysis of mesenchymal stem cell markers. Western blotting revealed the dose- and time-dependent downregulation of CD44 and CD105 (Fig. 1C-F), which are core hUC-MSC surface markers (Beeravolu N 2017). This attenuation of core mesenchymal markers correlated with morphological restructuring, suggesting a CPP-driven loss of stem cell identity.

### CPP Directs endothelial specification in human umbilical cord-derived MSCs

Concomitant with the CPP-induced attenuation of mesenchymal markers (CD44/CD105), we observed profound morphological restructuring in hUC-MSCs, characterized by spontaneous lacunar network formation reminiscent of nascent vasculature (Fig. 1B). This architectural transformation suggested CPP-driven endothelial differentiation.

**Fig. 2:**
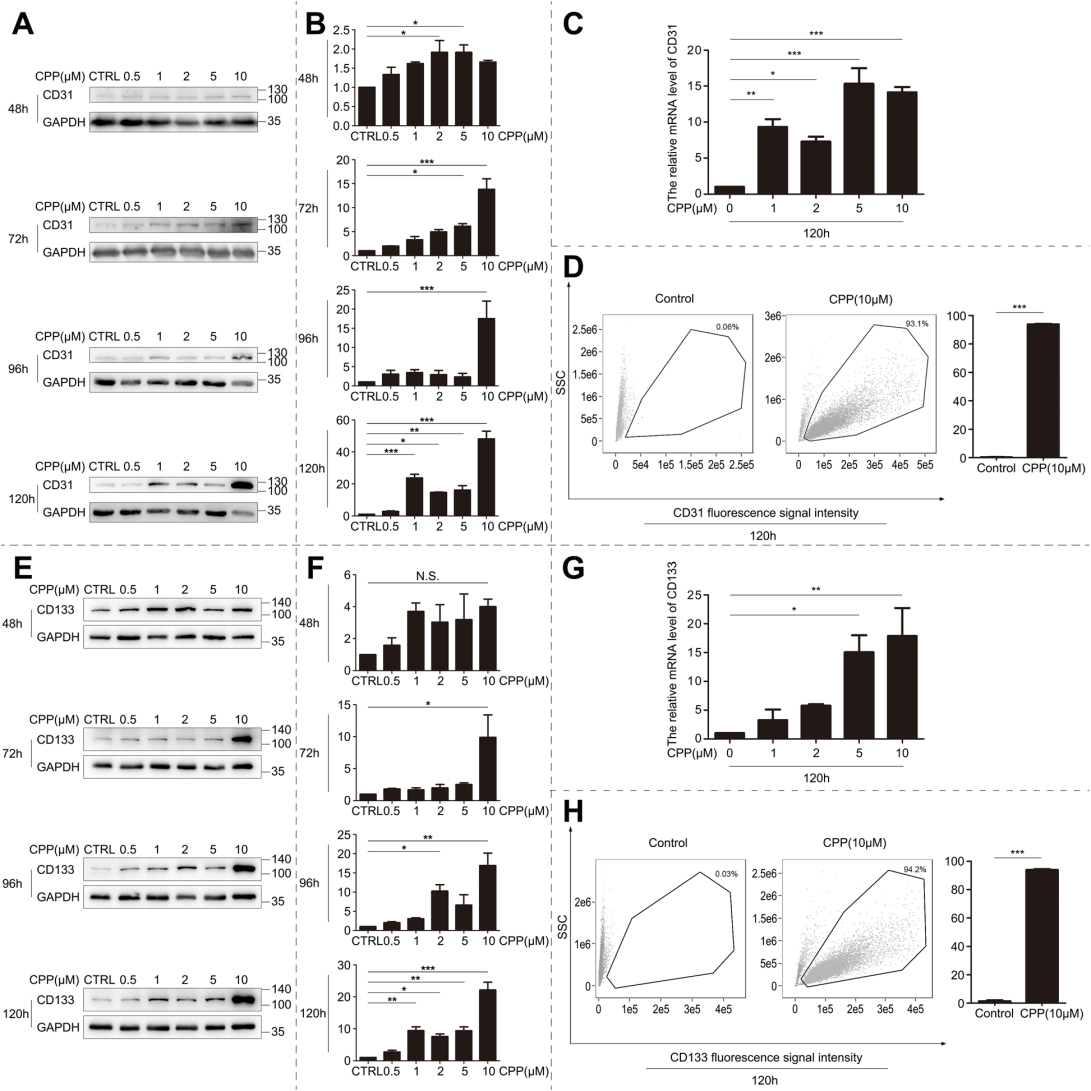
CPP Induces VEC Differentiation from UC-MSCs. **A** hUC-MSCs were treated with different concentrations of CPP (0,0.5, 1, 2, 5, 10 μM) for 48, 72, 96, and 120 h, and the changes in CD31 protein levels were detected by Western blot. **B** Quantification of CD31 Western blot bands using GAPDH as the loading control. **C** hUC-MSCs were treated with different concentrations of CPP (0, 1, 2, 5, 10 μM) for 120 h, and the mRNA level of CD31 was detected by qPCR. **D** hUC-MSCs were treated with different concentrations of CPP (0, 10 μM) for 120 h, and the proportion of CD31-positive cells was detected by flow cytometry. **E** hUC-MSCs were treated with different concentrations of CPP (0,0.5, 1, 2, 5, 10 μM) for 48, 72, 96, and 120 h, and the changes in CD133 protein levels were detected by Western blot. **F** Quantification of CD133 Western blot bands using GAPDH as the loading control. **G** hUC-MSCs were treated with different concentrations of CPP (0, 1, 2, 5, 10 μM) for 120 h, and the mRNA level of CD133 was detected by qPCR. **H** hUC-MSCs were treated with different concentrations of CPP (0, 10 μM) for 120 h, and the proportion of CD133-positive cells was detected by flow cytometry

To test this hypothesis, we quantified canonical endothelial determinants. We observed that the protein expression of CD31 (PECAM-1; mediates cell adhesion and vascular integrity) increased 4.2-fold (Fig. 2A, B) accompanied by a 3.8-fold increase in mRNA expression (Fig. 2C). Likewise, we detected that the protein levels of CD133 (transmembrane glycoprotein; maintains progenitor potency) increased 5.1-fold (Fig. 2E, F), paralleled by a 4.6-fold transcriptional upregulation (Fig. 2G). In addition, we observed a dose-dependent accumulation of VEGF-A (signaling protein; promotes angiogenesis) (Fig. S1B, C) and time-dependent upregulation of FGF2 (growth factor stimulates endothelial proliferation) (Fig. S1D, E).

Flow cytometry analysis confirmed terminal differentiation. We found that CD31⁺ cells expanded from 3.26% to 96.4% (29.6-fold; Fig. 2D). Likewise, CD133⁺ populations increased from 1.46% to 97.0% (66.4-fold; Fig. 2H). Finally, CD144⁺ (VE-cadherin; sustains vascular barrier function) subpopulations increased from 8.23% to 91.3% (11.1-fold; Fig. S1F). Collectively, these molecular and cellular transitions established a definitive CPP-driven endothelial specification.

### CPP-induced vascular endothelial cells exhibit native functionality

**Fig. 3:**
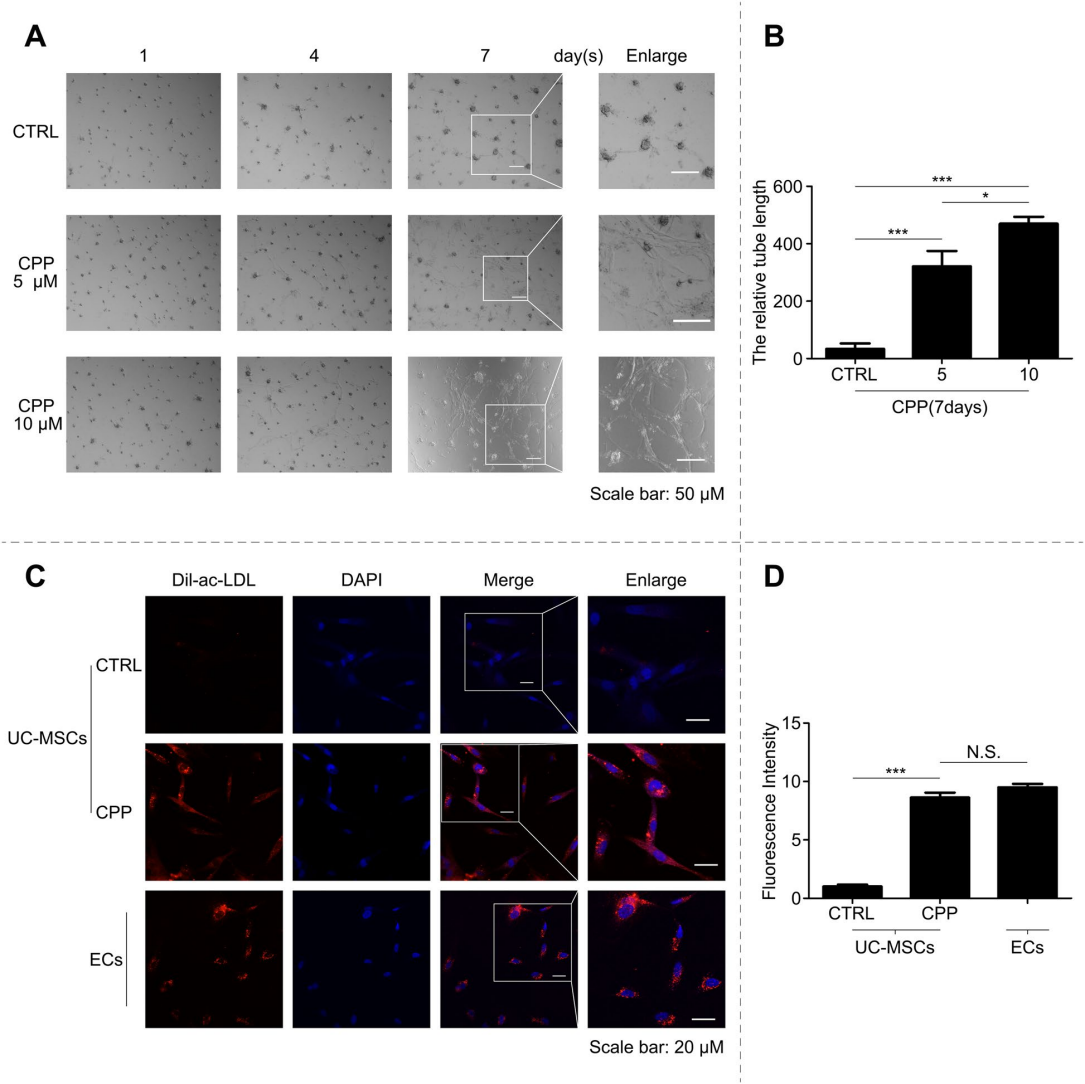
CPP-induced VECs are functional. **A** hUC-MSCs treated with different concentrations of CPP (0, 5, 10 μM) for 1, 4, and 7 days formed capillary-like tubes in Matrigel matrix gel, and representative images showing capillary morphogenesis were recorded at different time points. Scale bar: 50 μm. **B** The length of the tube on day 7 in (**A**) was analyzed using ImageJ and quantified for statistics. **C** hUC-MSCs were treated with 10 μM CPP for 120 h, and the phagocytosis of Dil-ac-LDL by cells was observed by stimulating Dil label fluorescence under a confocal microscope. Scale bar: 20 μm. **D** Fluorescence intensity statistics of hUC-MSCs treated/untreated with CPP and normal endothelial cells (HUVEC) group

To validate the functional maturity of CPP-treated VECs, we performed endothelial-specific assays. Matrigel-based lumenogenesis CPP-treated cells assembled extensive lumen-containing networks with a significantly elongated architecture, demonstrating robust angiogenic potential. This contrasted starkly with the compact cell clusters observed in untreated controls (Fig. 3A, B).

Moreover, Ac-LDL endocytic profiling revealed that CPP-derived VECs exhibited scavenger receptor activity indistinguishable from that of primary HUVECs (Fig. 3C, D), demonstrating preserved ligand-specific uptake.

These functional validations confirmed that CPP-derived VECs recapitulated essential endothelial activities, establishing their phenotypic and functional equivalence to native endothelia.

### Single-cell transcriptomics decodes functional endothelial heterogeneity during CPP induction

**Fig. 4:**
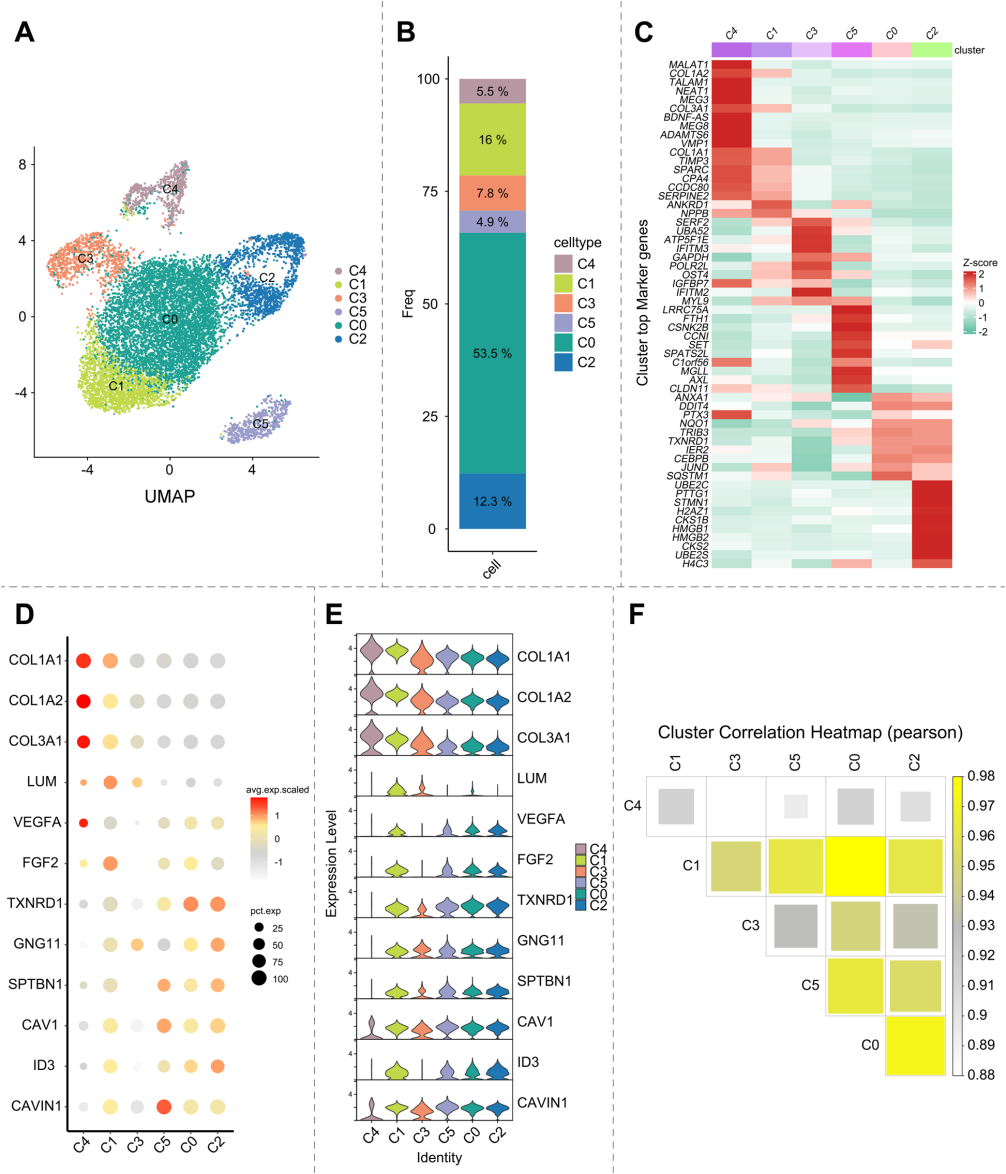
Single-cell transcriptomics deciphers the functional heterogeneity of VECs during CPP induction. **A** Each cluster classification is visualized as a UMAP plot. **B** Percentage of cells per computational cluster relative to total cells. **C** Heatmap depicting expression patterns of top marker genes across clusters. **D** Bubble plot visualizing highly expressed marker genes across cell clusters. **E** Violin plots visualize highly expressed marker genes across cell clusters. **F** Heatmap displaying Pearson correlation coefficients across cell clusters

To delineate the mechanistic basis of CPP-driven endothelial specification, we performed single-cell RNA sequencing (scRNA-seq) on hUC-MSCs treated with 10 μM CPP for 120 h. Using the 10 × Genomics platform, we captured 9851 high-quality cells and partitioned them into nine transcriptionally distinct clusters (Fig. 4A, B). The gene expression differences across these clusters are shown in heat maps (Fig. 4C). We annotated cell identities by mapping cluster-specific marker genes to established cell types. Cluster 4 exhibited high expression of mesenchymal cell markers (COL1A1, COL1A2, COL3A1, and LUM), designating it as a mesenchymal population (Fig. 4D, E). Notably, Gene Ontology analysis revealed significant enrichment of blood vessel development, morphogenesis, and angiogenesis terms in this cluster (Fig. S2), suggesting concomitant endothelial differentiation potential. Cluster 1 coexpressed mesenchymal and endothelial cell markers. Furthermore, Gene Ontology analysis revealed the concurrent enrichment of terms related to extracellular matrix organization (indicative of mesenchymal identity) and angiogenesis/blood vessel development (Fig. S3). These findings suggested that this cluster represents an intermediate cell state between the mesenchymal and endothelial lineages. Cluster 3 showed elevated expression of oxidative phosphorylation (OXPHOS)-associated genes (Fig. S4). Given that high OXPHOS activity typically reflects the basal metabolic state of a cell, such as active proliferation or heightened energy demand, rather than a specific differentiation trajectory or functional subtype, we designated this population as inter-OXPHOS cells. Clusters 5, 0, and 2 uniformly exhibited high expression of endothelial cell markers (including VEGFA, FGF2, TXNRD1, and GNG11) and were annotated as endothelial populations. Cluster 5 was strongly associated with actin cytoskeleton organization (Fig. S5), suggesting an activated endothelial state population that potentially engages in angiogenesis or inflammatory responses and requires cytoskeletal remodeling to support cell migration. Cluster 0 represented the dominant endothelial subpopulation, comprising 53.5% of all cells (Fig. 4B). Beyond expressing genes linked to basal proliferative/metabolic processes common to active cells, this cluster was uniquely enriched in nitric oxide (NO) biosynthesis pathways (Fig. S6). Given that endothelial cells are the primary producers of vasoactive NO, which regulates vasodilation, anti-inflammatory responses, and antithrombotic functions (Tousoulis et al. 2012; Tedgui and Mallat 2001), this finding confirmed that induced endothelial cells are highly active and retain their core physiological functions. In contrast, cluster 2 displayed signatures of proliferating endothelium and was markedly enriched in core cell division processes (Fig. S7). Pearson’s correlation analysis across clusters further validated the identification of distinct cell populations. Clusters 4 and 3 exhibited lower correlations with other clusters. Cluster 1, which represents an intermediate cell state, showed a higher correlation with all clusters. Whereas, clusters 0, 2, and 5, which all belonged to endothelial cells, demonstrated strong mutual correlations (Fig. 4F).

We have explicitly defined the endothelial cell population(s) by demonstrating the coordinated expression of multiple orthogonal endothelial programs within the same UMAP clusters. These programs include key receptors and signaling molecules (FLT1/VEGFR1, TEK/TIE2, NRP1/2) (Fig. S8A), endothelial transcription factors (ETS1, GATA2, KLF2, KLF4) (Fig. S8B), membrane and junctional components (CAV1, CAVIN1, PLXND1, ENG, TJP1) (Fig. S8C), and genes involved in endothelial function and metabolism (PROCR, NOS3/eNOS, EDN1, ACVRL1) (Fig. S8D). This is further supported by venous/arterial subtype signals (strong NR2F2, moderate EFNB2; low lymphatic PDPN) (Fig. S8E). The colocalization and enrichment of these diverse markers provide a clear, pathway-level definition of endothelial identity.

### Trajectory analysis identified MEG3 as a core regulator of endothelial transdifferentiation

**Fig. 5:**
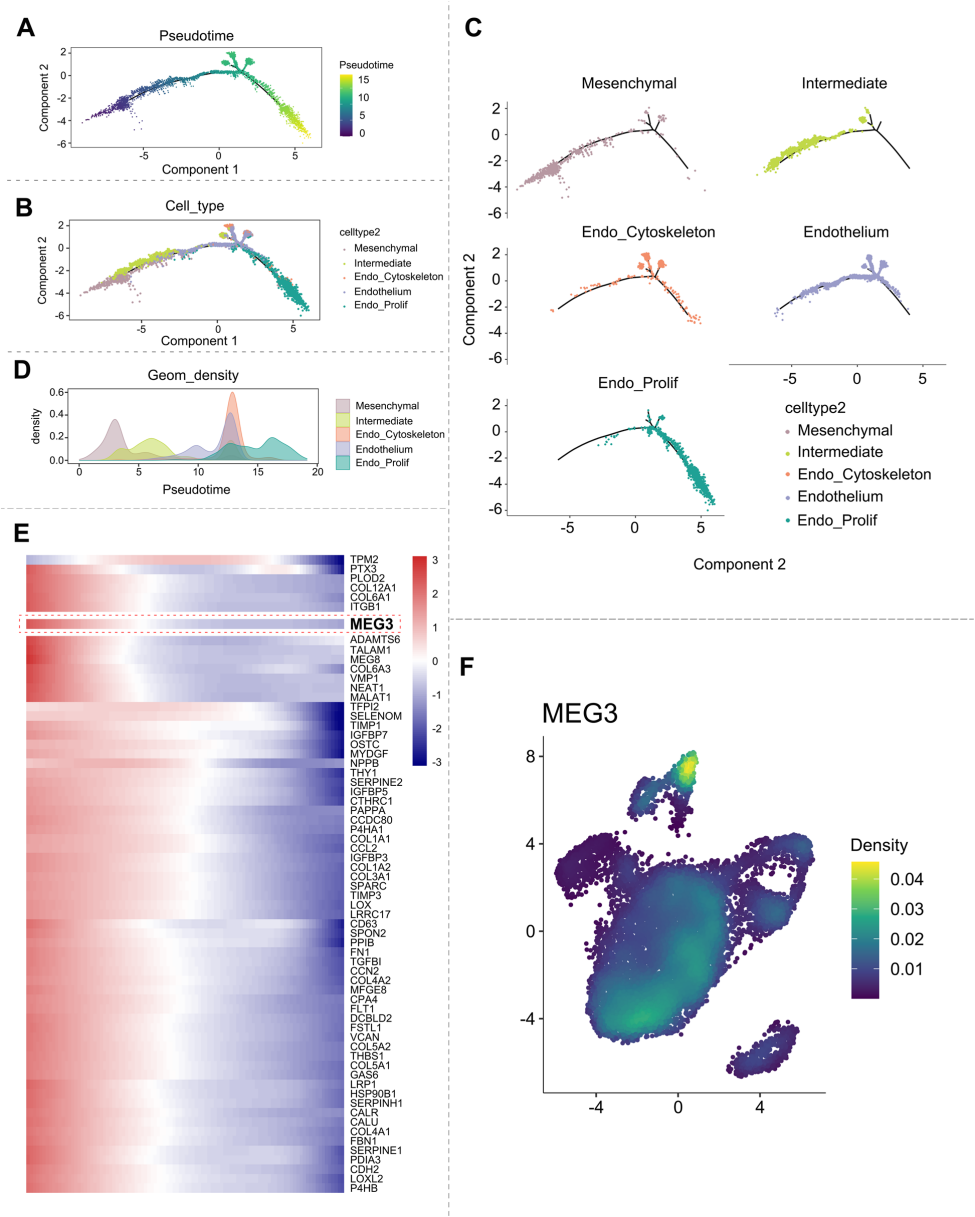
Trajectory analysis pinpointed MEG3 as a key regulator of endothelial transdifferentiation. **A** Visualization of subpopulation differentiation trajectories with pseudotime mapping. **B** Pseudotime-projected differentiation trajectories of functionally annotated cell types. **C** Spatial distribution of functionally annotated cell types along pseudotime-projected differentiation trajectories. **D** Probability density curves visualize cellular subpopulation distribution and state transitions through peak positioning. **E** Differential expression heatmap of genes downregulated along pseudotime trajectories. **F** Spatial density landscape of MEG3 expression on UMAP projection

Following CPP induction in hUC-MSCs, immunophenotyping revealed that > 70% of the cells underwent conversion to the endothelial lineage (Fig. S9A, B). Given that high OXPHOS cells typically represent terminally differentiated states and can obscure true biological trajectories, we excluded cluster 3 before pseudotemporal ordering (Fig. S9C). Subsequent trajectory analysis of the remaining clusters demonstrated a clear differentiation path from mesenchymal cells to endothelial populations through an intermediate state (Fig. 5A-C). Transcriptomic profiling confirmed significant divergence between endothelial-specific genes and those expressed in naive MSCs (Fig. 5D).

Therefore, we focused on the dynamically regulated genes along this trajectory to identify key regulators directly relevant to endothelial induction. Among the markedly downregulated genes, we identified the long noncoding RNA MEG3 as a candidate regulator (Fig. 5E). Notably, MEG3 promotes endothelial differentiation in murine adipose-derived stem cells via the miR-145-5p/KLF4 axis (Zhang et al. 2022). Concordantly, we observed pronounced MEG3 enrichment in mesenchymal cells and depletion in endothelial cells (Fig. 5F), suggesting its potential role as a key regulator of this transdifferentiation process.

### Loss-of-function validation established MEG3 as a decisive factor for endothelial commitment

**Fig. 6:**
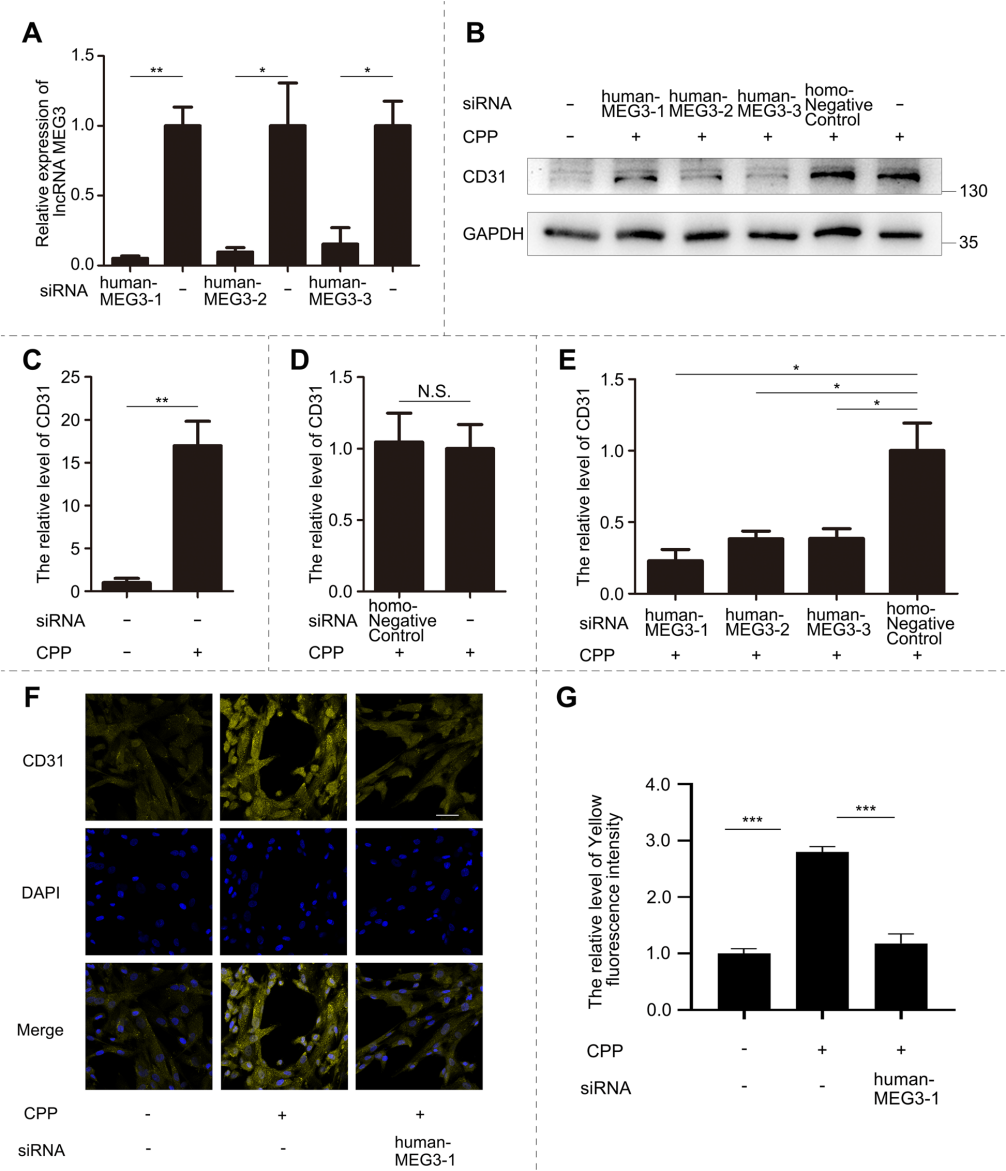
Loss-of-function analysis confirmed MEG3 is required for endothelial commitment. **A** After 48 h of interference with MEG3 by three kinds of siRNA, the levels of MEG3 were detected by qPCR and compared with those of the untreated group and statistically analyzed. **B** hUC-MSCs were treated with 10 μM CPP for 120 h, and different siRNAs were used to interfere with MEG3. The untreated group and the CPP-treated group were used as controls, and the ineffective siRNA group was added as a negative control. The changes in the protein level of CD31 were detected by Western blot. **C** The difference in CD31 protein expression between the untreated group and the CPP-treated group was statistically analyzed. **D** The difference in CD31 protein expression between the negative control group and the CPP-treated group was statistically analyzed. **E** The difference in CD31 protein expression between the MEG3 interference group and the negative control group was statistically analyzed. **F** hUC-MSCs were treated with 10 μM CPP for 120 h while subjected to siRNA-mediated MEG3 interference. Using the untreated group as control, CD31 protein was observed by confocal microscopy via immunofluorescence. Scale bar: 50 μm. **G** Statistical analysis of fluorescence intensity comparing CPP-untreated/treated groups with the CPP-treated plus siRNA interference group

To validate the regulatory role of MEG3 in endothelial differentiation, we designed three distinct siRNAs targeting different regions of MEG3. Using qPCR analysis, we confirmed the efficient MEG3 knockdown using all siRNAs (Fig. 6A). Next, we induced the endothelial differentiation of MEG3-depleted cells using CPP. We observed that the protein level of CD31 in the CPP-treated and negative control groups was significantly upregulated (Fig. 6B, C), with no significant differences observed between the two groups (Fig. 6D). The CD31 protein levels were significantly upregulated in both CPP-treated and nontargeted controls (Fig. 6B, C), with no significant differences between these conditions (Fig. 6D). Critically, all three MEG3-targeting siRNAs significantly suppressed CD31 protein expression compared with that in nontargeted controls (Fig. 6E). Consistently, immunofluorescence results confirmed that CD31 protein levels were markedly reduced upon MEG3 inhibition(Fig. 6F, G). These results established MEG3 as a decisive factor for the endothelial differentiation of hUC-MSCs, with its expression level dictating its cellular reprogramming capacity.

## Discussion

Over the past decade, various methodologies have been developed to differentiate stem cells into endothelial cells (ECs). These cells have been extensively tested in animal models of diverse human diseases, demonstrating their potential in regenerative medicine (Liu et al. 2018). Furthermore, the generation of numerous patient-specific stem cell lines has enabled the derivation of ECs for elucidating disease mechanisms (Menasché et al. 2015). The accumulating knowledge encompassing EC differentiation, functional assessment, and potential applications holds considerable promise for the development of novel strategies to combat cardiovascular disease.

In this context, our study identified the small-molecule compound CPP as a potent inducer of vascular endothelial cell (VEC) differentiation from human umbilical cord mesenchymal stem cells (hUC-MSCs). We confirmed this differentiation trajectory through rigorous assessment of lineage-specific marker proteins expressed in hUC-MSCs and induced VECs. The functional competence of CPP-derived VECs was unequivocally demonstrated using established assays; they exhibited robust angiogenic capabilities in vitro and efficiently internalized Dil-ac-LDL. Single-cell RNA sequencing provided further independent validation of the efficacy of CPP in driving the hUC-MSC-to-VEC transition.

Crucially, our investigation uncovered the long noncoding RNA MEG3 as a pivotal regulator of the CPP-induced differentiation pathway. Functional validation studies established that MEG3 is indispensable for the CPP-driven differentiation process.

Mesenchymal stem cells derived from the placenta have been demonstrated in a clinical study to restore the function of the ischemic tissue in patients with severe limb ischemia through immune regulation and angiogenesis induction. In this study, we induced hUC-MSCs to produce functional VECs using small chemical molecules, providing new ideas for studying the differentiation mechanism of VECs and a potential treatment for limb ischemic diseases. Considering the requirements of industrial production and drug applications, we employed a serum-free culture system with clear components for all cell culture and differentiation induction experiments. This system provided better data support and a process reference for the subsequent translation of research results into clinical studies.

Collectively, these findings provide experimental evidence that CPP is a promising lead compound for the development of novel therapeutics aimed at restoring vascular function. This study lays the foundation for future development of innovative strategies to treat pathologies involving vascular endothelial dysfunction.

## Materials and methods

### Antibodies

Antibodies against CD44 (A19020), CD105 (A19008), CD133 (A0818), CD31 (A2104) were from ABclonal Technology. Antibodies against VEGF-A (ab52917), FGF2 (ab208687) were from Abcam. The antibody against GAPDH(G9545) was from Sigma-Aldrich. Horseradish peroxidase-conjugated secondary antibodies were from Jackson Immunoresearch. PE anti-human CD31, PE anti-human CD133, and PE anti-human CD144 antibodies used for flow cytometry were from Biolegend.

### Cell culture

The human umbilical cord-derived mesenchymal stem cells (hUC-MSCs) were obtained from the Shandong Cell and Tissue Bank. The procurement and use of these cells for this research were in full compliance with the ethical guidelines of the bank and were approved by its institutional ethical committee. All donor mothers provided written informed consent for the use of the umbilical cord tissues for scientific research. Cells were cultured in Mesenchymal Stem Cell Basal Medium (SuperCulture) supplemented with 10% EliteGro™-Advanced MSC Serum Replacement (EliteCell). hUC-MSCs were cultured in a humidified incubator at 37 °C in a 5% CO_2_ atmosphere. Cells were seeded in appropriate dishes (35,000 cells/ml), and all cell lines were authenticated by DNA short tandem repeat (STR) profiling and were confirmed to be mycoplasma negative.

### Cell morphology

Morphological changes of hUC-MSCs were examined using an inverted phase contrast microscope (Eclipse TS-100, Nikon) after 120 h of treatment with CPP at the indicated concentrations.

### Western blot analysis

Cell lysates (30 μg protein per lane) were separated by SDS-PAGE, after which the proteins were transferred to polyvinylidene difluoride membranes. At room temperature, the membranes were blocked with 5% non-fat milk in TBST (TBS containing 0.05% Tween-20) for 1 h. After that, the membranes were incubated with the primary antibody at 4 °C overnight, then were washed with TBST three times for 5 min each. Each membrane was incubated with the secondary antibody at room temperature for 1 h, and then washed with TBST 3 times, for 5 min each time. Antibodies bound to proteins were detected using an enhanced chemiluminescence detection kit (34,080, Thermo Fisher). Relative quantities of specific bands were analyzed by Image J software and were normalized to loading controls.

### Quantitative real-time PCR

RNA was extracted from the whole-cell fraction by the Trizol reagent method (Takara), and extracted total RNAs were reverse transcribed using the primer sequences of the target genes. Reverse transcription was performed using the PrimeScript RT reagent kit with gDNA Eraser (Takara). PCR reactions involved the use of SYBR Premix Ex Taq (Tli RNaseH Plus, Takara) and the levels of expressed genes were measured using MxPro 4.00 (Stratagene). The following primers were used:

- CD31: 5’-TCAGACGTGCAGTACACGGA-3’ (forward) and 5’-CTTTCCACGGCATCAGGGAC-3’ (reverse);
- CD133: 5’-GTGGCGTGTGCGGCTATGAC-3’ (forward) and 5’-CCAACTCCAACCATGAGGAAGACG-3’ (reverse);
- MEG3: 5’-CTGCCCATCTACACCTCACG-3’ (forward) and 5’-CTCTCCGCCGTCTGCGCTAGGGGCT-3’ (reverse);
- β-Actin: 5’-CCTGGCACCCAGCACAAT-3’ (forward) and 5’-GCCGATCCACACGGAGTACT-3’ (reverse).

### Flow cytometry

hUC-MSCs were treated with 0.1% DMSO (as a control) or with CPP for 120 h. Next, cells were digested into single cells by 0.25% trypsin (Sangon Biotech) and collected into 15-mL centrifuge tubes. Centrifugation at 300 g for 15 min was performed, then the supernatant was discarded. Cells were washed twice with 1 × PBS and suspended in 1 × PBS supplemented with 2% (v/v) FBS by centrifugation at 300 g for 15 min each time. We discarded the supernatant. Cells were resuspended in 1 × PBS with 2% (v/v) FBS and incubated at 4℃ for 1 h with antibodies as indicated below. After staining, the cells were analyzed on a flow cytometer (ImageStreamX MarkII, Merck).

### Matrigel assays

After mixing the culture medium and Matrigel (Corning Matrigel Basement Membrane Matrix, Corning) at a ratio of 1:3, 300 μl of Matrigel was added to each well of a 24-well plate. The 24-well plate was incubated in a humidified incubator at 37 °C and 5% CO_2_ for 30 min. The cells were trypsinized, resuspended in culture medium, and seeded in a 24-well plate at a concentration of 4 × 10,000 cells/ml. The morphological changes of hUC-MSCs were observed using an inverted phase contrast microscope (Eclipse TS-100, Nikon). The tubule length was analyzed using Image J software and normalized to the control group.

### Acetylated-LDL uptake assay

hUC-MSCs were treated with 0.1% DMSO (as a control) or with CPP for 120 h, cells were incubated with DiI-Ac-LDL (L3484, Invitrogen) at 10 μg/ml in growth media for 4 h. Next, cells were fixed with 4% Paraformaldehyde (w/v) at room temperature for 20 min and washed with 1 × PBS three times. Finally, cells were rinsed and stained with DAPI and monitored by a laser scanning confocal microscope (Zeiss, Germany). The fields of view were randomly selected and the fluorescence intensity was counted.

### Single-cell RNA sequencing library construction

After hUC-MSCs were treated with 10 μM CPP for 120 h, trypsin was used to digest the cells into single cells and counted. A small amount of single cell suspension was taken, an equal volume of 0.4% trypan blue dye was added, the cells were counted, and the concentration of live cells was adjusted to the ideal range (1000–2000/μl). Gel beads containing barcode information were mixed with cells and enzymes to form Gel Beads-In-Emulsions (GEMs). Subsequently, the gel beads were dissolved to release the capture sequence containing the barcode sequence, reverse transcribed cDNA fragments, and the samples were labeled. Next, the gel beads were broken and the oil droplets were broken, and PCR amplification was performed using cDNA as a template. The products of all GEMs were mixed to construct a standard sequencing library. The cDNA was then cut into 200–300 bp fragments, followed by end repair, A-tailing, ligation of sequencing adapters P5, P7 and sample index, and finally PCR amplification to obtain a DNA library (Fig. S1).

### Single-cell RNA sequencing data analysis

Raw reads were processed using the 10X CellRanger pipeline to align these to the reference transcriptome (*Homo sapiens*) and to generate gene-cell count matrices. Initial quality control and clustering were performed with the aid of Seurat (Butler et al. 2018). To remove dead or misidentified cells as well as doublets (Fig. S2), cells with a restricted number of genes between 330 and 4700, a restricted total number of UMIs below 23,000, and a percentage of reads mapping to mitochondrial genes exceeding 10% were excluded from further analysis (Figs. S3, S4).

Pseudotime trajectories of differentiation were generated using the Monocle R Package Pseudotime analysis proceeds on the basis that cells undergo biological processes in an asynchronous manner, and thus that cells can be ordered along a calculated trajectory to infer the transcriptional changes throughout the process (Trapnell et al. 2014). Genes that were most differentially expressed in identified clusters were used to assign pseudotime values to individual cells. Differentially expressed genes across pseudotime were identified using the differentialGeneTest command in Monocle. RNA velocity of individual cells was calculated using the Velocyto R package. RNA velocity analysis determined the fraction of spliced-to-unspliced reads to predict the future transcriptional state of individual cells.

All further experimental and analytical details are included in the Supplementary Materials.

### RNA interference experiments

Cells were seeded in 6-well plates and transfected when the cell density reached 60%−80%. 5 μl siRNA was diluted with 250 μl Opti-MEM. 5 μl lipofectamine 3000 (Thermo Fisher) was diluted with 250 μl Opti-MEM. After siRNA and lipofectamine 3000 were placed on ice for 5 min, they were mixed by pipetting and placed on ice for 15 min. 1500 μl serum-free medium was added to the transfection mixture and then added to the 6-well plate. After 4–6 h of transfection, fresh complete medium was replaced and CPP at an appropriate concentration was added. The above steps were repeated every 48 h. After continuous interference for 120 h, protein and RNA were extracted for subsequent experiments. The RNA interference sequence is as follows:

- Human-MEG3-1: GAUGCGGUUCCAAAGCACA(dT)(dT) (sense), UGUGCUUUGGAACCGCAUC(dT)(dT) (antisense);
- Human-MEG3-2: GCUCAUACUUUGACUCUAU(dT)(dT) (sense), AUAGAGUCAAAGUAUGAGC(dT)(dT) (antisense);
- Human-MEG3-3: CAUGCUACUGAAUCACCAA(dT)(dT) (sense), UUGGUGAUUCAGUAGCAUG(dT)(dT) (antisense).

### Statistical analysis

At least three independent replicate experiments were performed, and statistical analysis was performed using Graphpad Prism. One-way ANOVA test was used to analyze the differences between groups. If only two groups were compared, Student’s t-test was used. The results are expressed in the form of mean + SEM, * *p* < 0.05 represents a significant difference, ** *p* < 0.01, *** *p* < 0.001 represents a very significant difference, N.S.: *p* > 0.05 represents no significant difference. Randomly select image fields, images were processed by GraphPad Prism 5 and Adobe Photoshop CC 2015.

## Supplementary Information

Supplementary Material 1. Supplementary Figures 1~9.

## Data Availability

The single cell datasets of this study have been deposited into the China National GeneBank Sequence Archive (CNSA) with accession number CNP0008689.

## Abbreviations

hUC-MSCs: Human umbilical cord mesenchymal stem cells
VECs: Vascular endothelial cells
EPCs: Endothelial progenitor cells
scRNA-seq: Single-cell RNA sequencing
CVDs: Cardiovascular diseases
CLI: Critical limb ischemia
OXPHOS: Oxidative phosphorylation
NO: Nitric oxide
ECs: Endothelial cells
EB: Embryoid Body

## References

[CR1] Adams WJ, Zhang Y, Cloutier J, Kuchimanchi P, Newton G, Sehrawat S, et al. Functional vascular endothelium derived from human induced pluripotent stem cells. Stem Cell Rep. 2013;1(2):105–13. 10.1016/j.stemcr.2013.06.007. 10.1016/j.stemcr.2013.06.007 PMC3757754 24052946

[CR2] Bai H, Xie YL, Gao YX, Cheng T, Wang ZZ. The balance of positive and negative effects of TGF-β signaling regulates the development of hematopoietic and endothelial progenitors in human pluripotent stem cells. Stem Cells Dev. 2013;22(20):2765–76. 10.1089/scd.2013.0008. 10.1089/scd.2013.0008 PMC3787486 23758278

[CR3] Beeravolu N, McKee C, Alamri A, Mikhael S, Brown C, Perez-Cruet M, et al. Isolation and Characterization of Mesenchymal Stromal Cells from Human Umbilical Cord and Fetal Placenta. J Vis Exp. 2017(122). 10.3791/55224. 10.3791/55224 PMC5564456 28447991

[CR4] Butler A, Hoffman P, Smibert P, Papalexi E, Satija R. Integrating single-cell transcriptomic data across different conditions, technologies, and species. Nat Biotechnol. 2018;36(5):411–20. 10.1038/nbt.4096. 10.1038/nbt.4096 PMC6700744 29608179

[CR5] Choi KD, Yu J, Smuga-Otto K, Salvagiotto G, Rehrauer W, Vodyanik M, et al. Hematopoietic and endothelial differentiation of human induced pluripotent stem cells. Stem Cells. 2009;27(3):559–67. 10.1038/s41587-024-02360-7. 10.1634/stemcells.2008-0922 PMC2931800 19259936

[CR6] Global, regional, and national age-sex specific all-cause and cause-specific mortality for 240 causes of death, 1990–2013: a systematic analysis for the Global Burden of Disease Study 2013. Lancet. 2015;385(9963):117–71. 10.1016/S0140-6736(14)61682-2. 10.1016/S0140-6736(14)61682-2 PMC4340604 25530442

[CR7] Golchin A, Chatziparasidou A, Ranjbarvan P, Niknam Z, Ardeshirylajimi A. Embryonic Stem Cells in Clinical Trials: Current Overview of Developments and Challenges. Adv Exp Med Biol. 2021;1312:19–37. 10.1007/5584_2020_592. 10.1007/5584_2020_592 33159303

[CR8] Gupta R, Losordo DW. Cell therapy for critical limb ischemia: moving forward one step at a time. Circ Cardiovasc Interv. 2011;4(1):2–5. 10.1161/CIRCINTERVENTIONS.110.960716. 10.1161/CIRCINTERVENTIONS.110.960716 PMC3123778 21325196

[CR9] Howard DP, Banerjee A, Fairhead JF, Hands L, Silver LE, Rothwell PM. Population-based study of incidence, risk factors, outcome, and prognosis of ischemic peripheral arterial events: implications for prevention. Circulation. 2015;132(19):1805–15. 10.1186/s13054-016-1208-6. 10.1161/CIRCULATIONAHA.115.016424 PMC4633967 26350058

[CR10] Huang Y, Hao X, Lin Z, Li L, Jiang H, Zhang H, et al. Bio-distribution and toxicity potential of human umbilical cord mesenchymal stem cells in cynomolgus monkeys. Sci Rep. 2024;14(1):12251. 10.1038/s41598-024-63118-4. 10.1038/s41598-024-63118-4 PMC11133417 38806615

[CR11] Khan FA, Almohazey D, Alomari M, Almofty SA. Isolation, culture, and functional characterization of human embryonic stem cells: current trends and challenges. Stem Cells Int. 2018;2018:1429351. 10.1155/2018/1429351. 10.1155/2018/1429351 PMC6142731 30254679

[CR12] Li T, Xia M, Gao Y, Chen Y, Xu Y. Human umbilical cord mesenchymal stem cells: an overview of their potential in cell-based therapy. Expert Opin Biol Ther. 2015;15(9):1293–306. 10.1517/14712598.2015.1051528. 10.1517/14712598.2015.1051528 26067213

[CR13] Li Z, Gao J, Sun D, Jiao Q, Ma J, Cui W, et al. LncRNA MEG3: potential stock for precision treatment of cardiovascular diseases. Front Pharmacol. 2022;13:1045501. 10.3389/fphar.2022.1045501. 10.3389/fphar.2022.1045501 PMC9744949 36523500

[CR14] Liew A, Bhattacharya V, Shaw J, Stansby G. Cell therapy for critical limb ischemia: a meta-analysis of randomized controlled trials. Angiology. 2016;67(5):444–55. 10.1177/0003319715595172. 10.1177/0003319715595172 26195561

[CR15] Liu LP, Zheng YW. Predicting differentiation potential of human pluripotent stem cells: possibilities and challenges. World J Stem Cells. 2019;11(7):375–82. 10.4252/wjsc.v11.i7.375. 10.4252/wjsc.v11.i7.375 PMC6682503 31396366

[CR16] Liu HB, Gong YF, Yu CJ, Sun YY, Li XY, Zhao D, et al. Endothelial progenitor cells in cardiovascular diseases: from biomarker to therapeutic agent. Regen Med Res. 2013;1(1):9. 10.1186/2050-490X-1-9. 10.1186/2050-490X-1-9 PMC4430916 25984328

[CR17] Liu YW, Chen B, Yang X, Fugate JA, Kalucki FA, Futakuchi-Tsuchida A, et al. Human embryonic stem cell-derived cardiomyocytes restore function in infarcted hearts of non-human primates. Nat Biotechnol. 2018;36(7):597–605. 10.1038/nbt.4162. 10.1038/nbt.4162 PMC6329375 29969440

[CR18] Menasché P, Vanneaux V, Hagège A, Bel A, Cholley B, Cacciapuoti I, et al. Human embryonic stem cell-derived cardiac progenitors for severe heart failure treatment: first clinical case report. Eur Heart J. 2015;36(30):2011–7. 10.1093/eurheartj/ehv189. 10.1093/eurheartj/ehv189 25990469

[CR19] Mushahary D, Spittler A, Kasper C, Weber V, Charwat V. Isolation, cultivation, and characterization of human mesenchymal stem cells. Cytometry A. 2018;93(1):19–31. 10.1002/cyto.a.23242. 10.1002/cyto.a.23242 29072818

[CR20] O'Neill TJt, Wamhoff BR, Owens GK, Skalak TC. Mobilization of bone marrow-derived cells enhances the angiogenic response to hypoxia without transdifferentiation into endothelial cells. Circ Res. 2005;97(10):1027–35. 10.1161/01.RES.0000189259.69645.25. 10.1161/01.RES.0000189259.69645.25 16210550

[CR21] Pacinella G, Ciaccio AM, Tuttolomondo A. Endothelial dysfunction and chronic inflammation: the cornerstones of vascular alterations in age-related diseases. Int J Mol Sci. 2022;23(24):15722. 10.3390/ijms232415722. 10.3390/ijms232415722 PMC9779461 36555364

[CR22] Peripheral arterial disease in people with diabetes. Diabetes Care. 2003;26(12):3333–41. 10.2337/diacare.26.12.3333. 10.2337/diacare.26.12.3333 14633825

[CR23] Qin H, Zhao A, Fu X. Small molecules for reprogramming and transdifferentiation. Cell Mol Life Sci. 2017;74(19):3553–75. 10.1007/s00018-017-2586-x. 10.1007/s00018-017-2586-x PMC11107793 28698932

[CR24] Rufaihah AJ, Huang NF, Kim J, Herold J, Volz KS, Park TS, et al. Human induced pluripotent stem cell-derived endothelial cells exhibit functional heterogeneity. Am J Transl Res. 2013;5(1):21–35. PMC3560482 23390563

[CR25] Sahara M, Hansson EM, Wernet O, Lui KO, Später D, Chien KR. Manipulation of a VEGF-Notch signaling circuit drives formation of functional vascular endothelial progenitors from human pluripotent stem cells. Cell Res. 2014;24(7):820–41. 10.1038/cr.2014.59. 10.1038/cr.2014.59 PMC4085760 24810299

[CR26] Tedgui A, Mallat Z. Anti-inflammatory mechanisms in the vascular wall. Circ Res. 2001;88(9):877–87. 10.1161/hh0901.090440. 10.1161/hh0901.090440 11348996

[CR27] Todtenhaupt P, Franken LA, Groene SG, van Hoolwerff M, van der Meeren LE, van Klink JMM, et al. A robust and standardized method to isolate and expand mesenchymal stromal cells from human umbilical cord. Cytotherapy. 2023;25(10):1057–68. 10.1016/j.jcyt.2023.07.004. 10.1016/j.jcyt.2023.07.004 37516948

[CR28] Tompkins BA, Balkan W, Winkler J, Gyöngyösi M, Goliasch G, Fernández-Avilés F, et al. Preclinical studies of stem cell therapy for heart disease. Circ Res. 2018;122(7):1006–20. 10.1161/CIRCRESAHA.117.312486. 10.1161/CIRCRESAHA.117.312486 PMC7340290 29599277

[CR29] Tousoulis D, Kampoli A-M, Tentolouris N, Papageorgiou C, Stefanadis C. The role of nitric oxide on endothelial function. Curr Vasc Pharmacol. 2012;10(1):4–18. 10.2174/157016112798829760. 10.2174/157016112798829760 22112350

[CR30] Trapnell C, Cacchiarelli D, Grimsby J, Pokharel P, Li S, Morse M, et al. The dynamics and regulators of cell fate decisions are revealed by pseudotemporal ordering of single cells. Nat Biotechnol. 2014;32(4):381–6. 10.1038/nbt.2859. 10.1038/nbt.2859 PMC4122333 24658644

[CR31] Woodruff RC, Tong X, Khan SS, Shah NS, Jackson SL, Loustalot F, et al. Trends in cardiovascular disease mortality rates and excess deaths, 2010–2022. Am J Prev Med. 2024;66(4):582–9. 10.1016/j.amepre.2023.11.009. 10.1016/j.amepre.2023.11.009 PMC10957309 37972797

[CR32] Xie X, Fu Y, Liu J. Chemical reprogramming and transdifferentiation. Curr Opin Genet Dev. 2017;46:104–13. 10.1016/j.gde.2017.07.003. 10.1016/j.gde.2017.07.003 28755566

[CR33] Xu Q, Hou W, Zhao B, Fan P, Wang S, Wang L, et al. Mesenchymal stem cells lineage and their role in disease development. Mol Med. 2024;30(1):207. 10.1186/s10020-024-00967-9. 10.1186/s10020-024-00967-9 PMC11552129 39523306

[CR34] Zhang L-J, Zhao X, Yang D, Jia Z-Z, Han X, Sun L-Q, et al. A new water-soluble and mitochondria-targeted fluorescence probe for ratiometric detection of hypochlorous acid in living cells. Sens Actuators B Chem. 2018;276:8–12. 10.1016/j.snb.2018.08.071.

[CR35] Zhang H, Liu G, Mao X, Yang L, Wang B, Yuan X. LncRNA MEG3 induces endothelial differentiation of mouse derived adipose-derived stem cells by targeting MiR-145-5p/KLF4. Mol Biol Rep. 2022;49(9):8495–505. 10.1007/s11033-022-07671-z. 10.1007/s11033-022-07671-z 35802277

[CR36] Zhang P, Dong B, Yuan P, Li X. Human umbilical cord mesenchymal stem cells promoting knee joint chondrogenesis for the treatment of knee osteoarthritis: a systematic review. J Orthop Surg Res. 2023;18(1):639. 10.1186/s13018-023-04131-7. 10.1186/s13018-023-04131-7 PMC10466768 37644595

