# Supplementary material for: Discovery of a chemical small molecule inducing umbilical cord mesenchymal stem cell differentiation to vascular endothelial cells
Source: Cell Regen. 2026 Jan 16;15:2. doi: 10.1186/s13619-025-00278-2 (PMC12811175; doi:10.1186/s13619-025-00278-2)
Supplement: Supplementary file 1 — Supplementary Material 1. Supplementary Figures 1~9. [file 13619_2025_278_MOESM1_ESM.docx]

**Supplementary Figure**


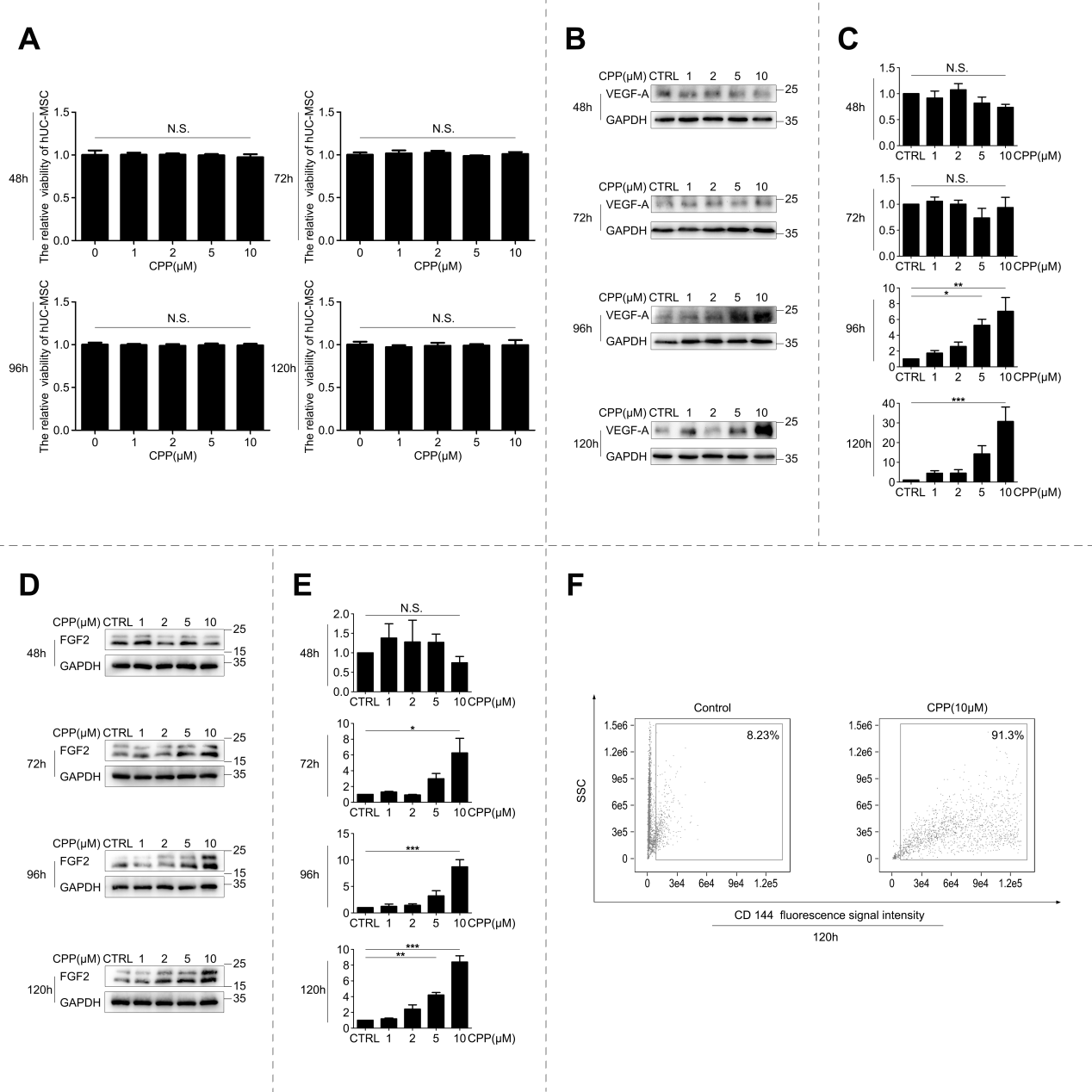


**Supplementary Figure 1** (A) hUC -MSCs were treated with different concentrations of CPP (0, 1, 2, 5, 10 μM) for 48, 72, 96, and 120 hours, and cell viability was determined using CCK-8. (B) hUC-MSCs were treated with different concentrations of CPP (0, 1, 2, 5, 10 μM) for 48, 72, 96, and 120 hours, and the changes in VEGF-A protein levels were detected by Western blot. (C) Quantification of VEGF-A Western blot bands using GAPDH as the loading control. (D) hUC-MSCs were treated with different concentrations of CPP (0, 1, 2, 5, 10 μM) for 48, 72, 96, and 120 hours, and the changes in FGF-2 protein levels were detected by Western blot. (E) Quantification of FGF-2 Western blot bands using GAPDH as the loading control. (F) hUC-MSCs were induced with different concentrations of CPP (0, 10 μM) for 120 h, and the proportion of CD144-positive cells was detected by flow cytometry.


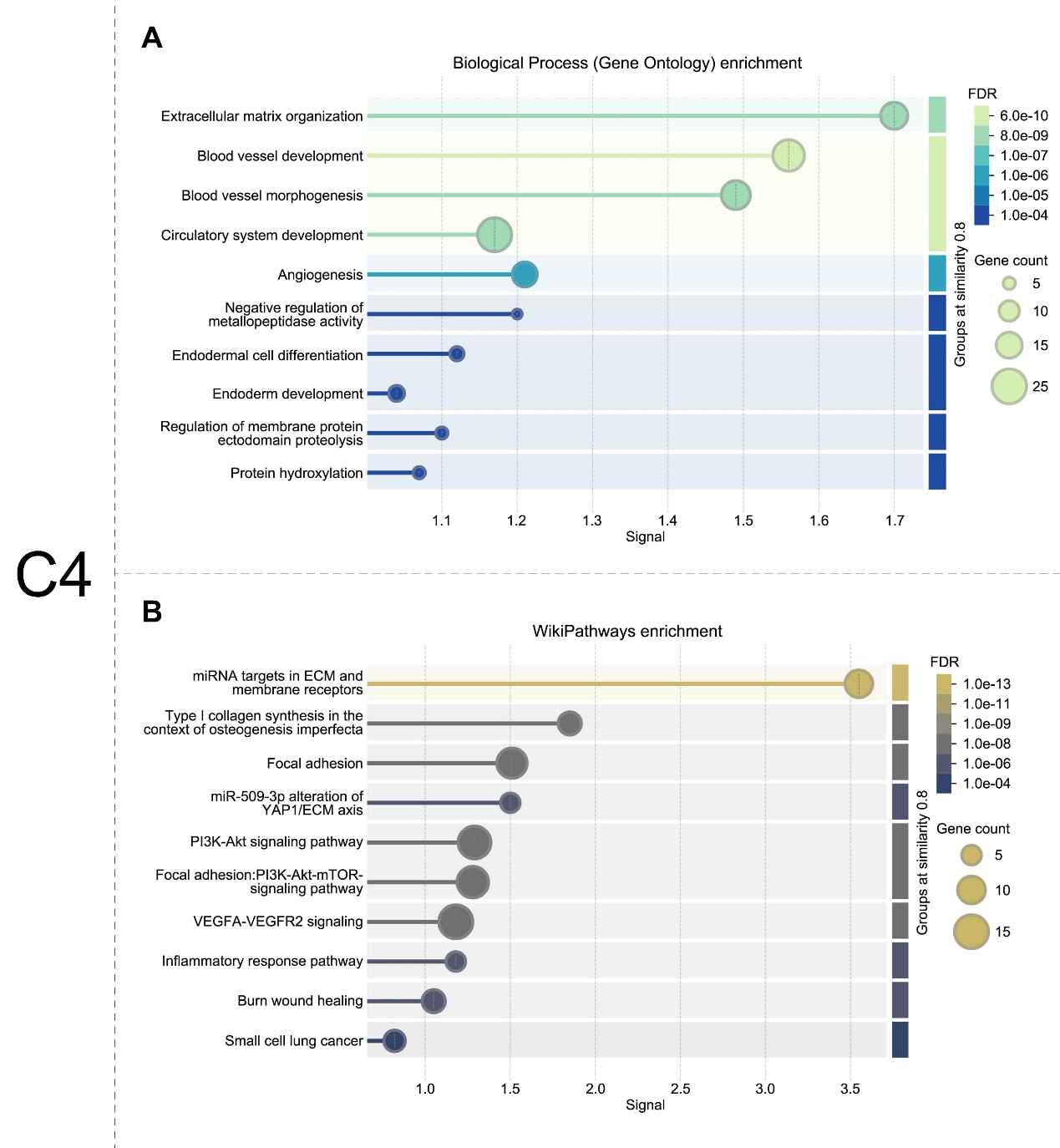


**Supplementary Figure 2** (A) Gene Ontology biological process enrichment in mesenchymal subcluster C4. (B) WikiPathways enrichment analysis in mesenchymal subcluster C4.


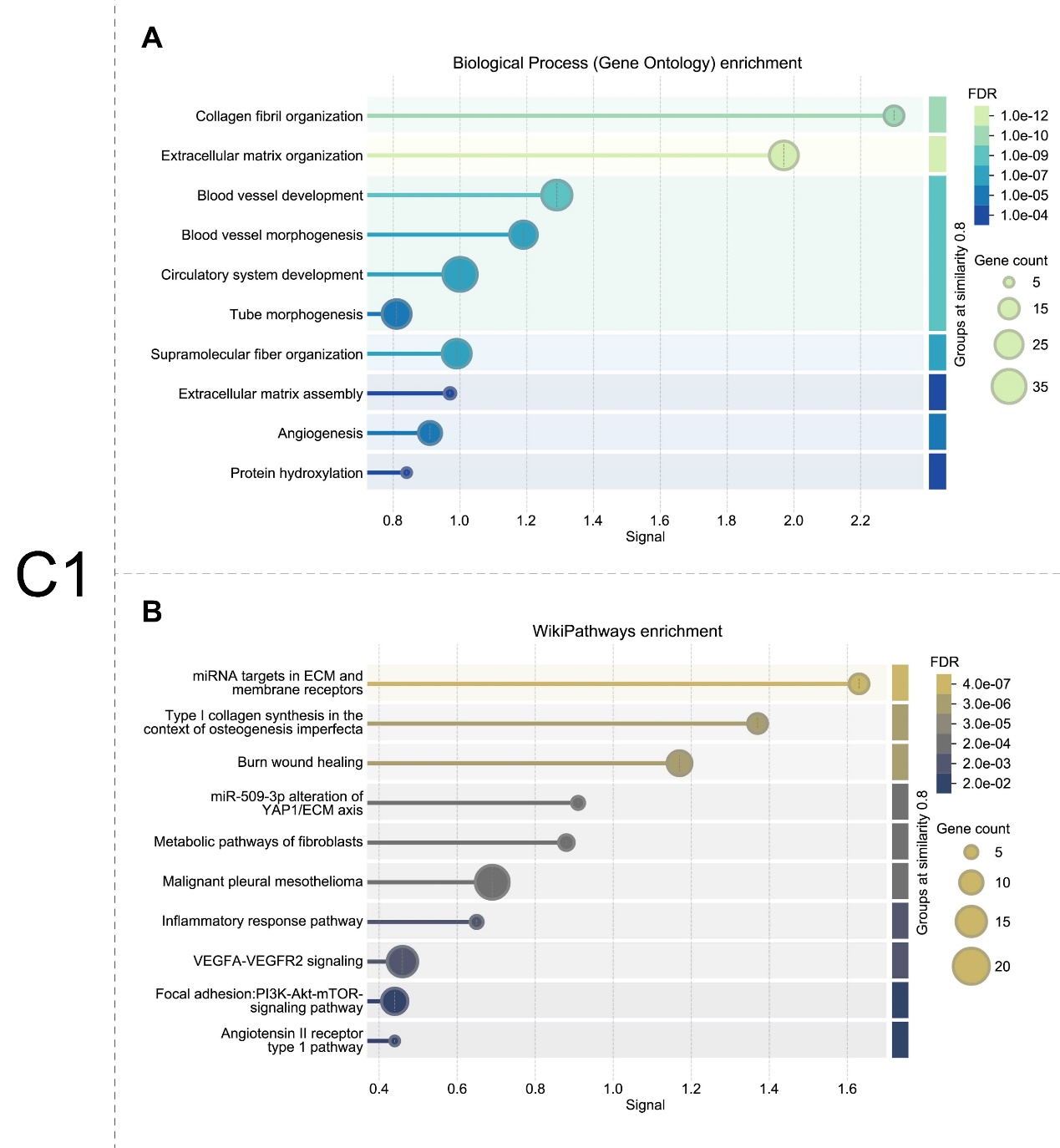


**Supplementary Figure 3** (A) Gene Ontology biological process enrichment analysis of transitional mesenchymal-endothelial subcluster C1. (B) WikiPathways enrichment in transitional mesenchymal-endothelial subcluster C1.


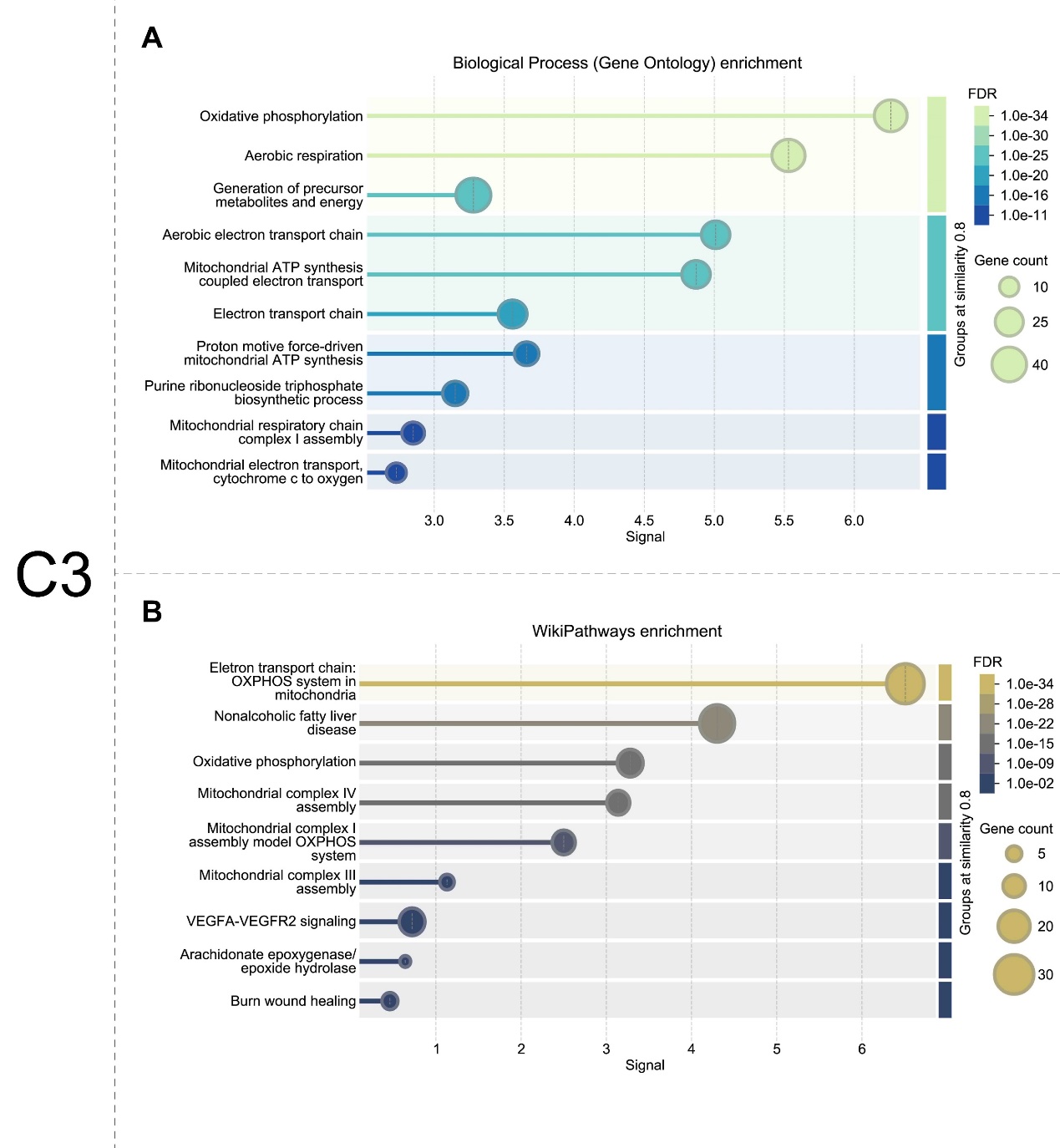


**Supplementary Figure 4** (A) Gene Ontology biological process enrichment in high-OXPHOS subcluster C3. (B) WikiPathways enrichment in high-OXPHOS subcluster C3.


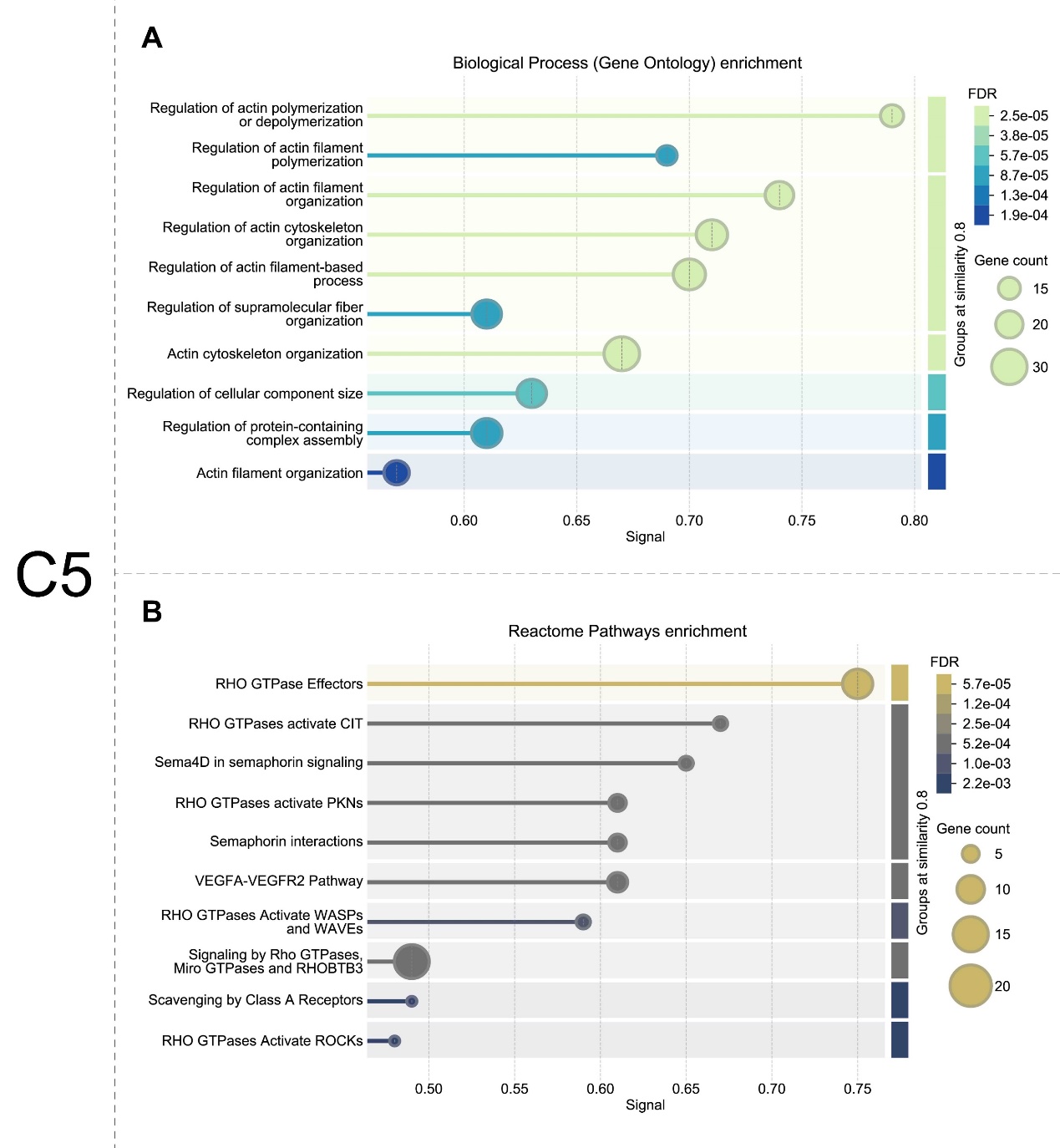


**Supplementary Figure 5** (A) Gene Ontology biological process enrichment in activated endothelial subcluster C5. (B) WikiPathways enrichment in activated endothelial subcluster C5.


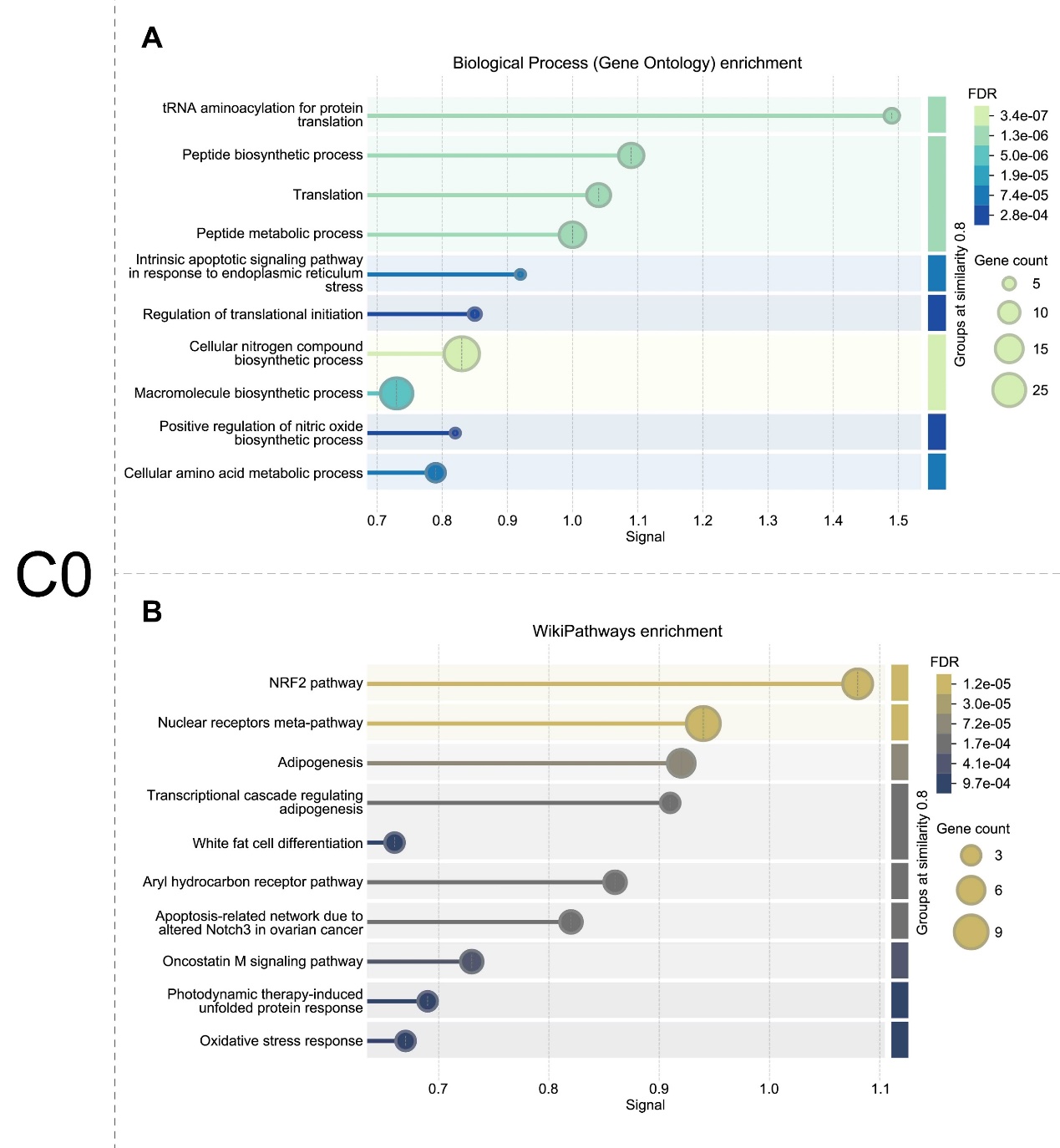


**Supplementary Figure 6** (A) Gene Ontology biological process enrichment analysis of endothelial subcluster C0. (B) WikiPathways enrichment analysis plot for endothelial subcluster C0.


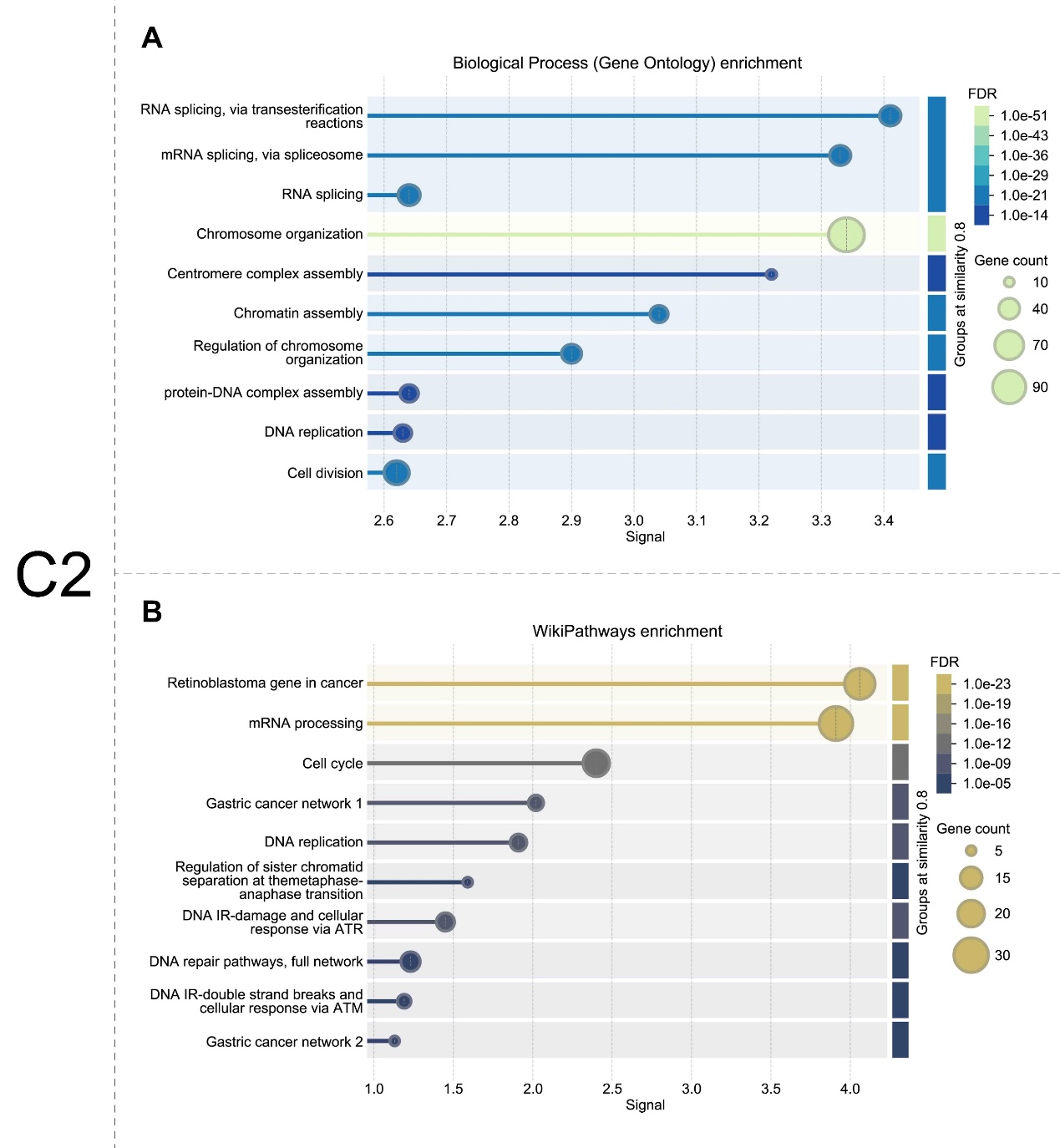


**Supplementary Figure 7** (A) Gene Ontology biological process enrichment in proliferative endothelial subcluster C2. (B) WikiPathways enrichment in proliferative endothelial subcluster C2.


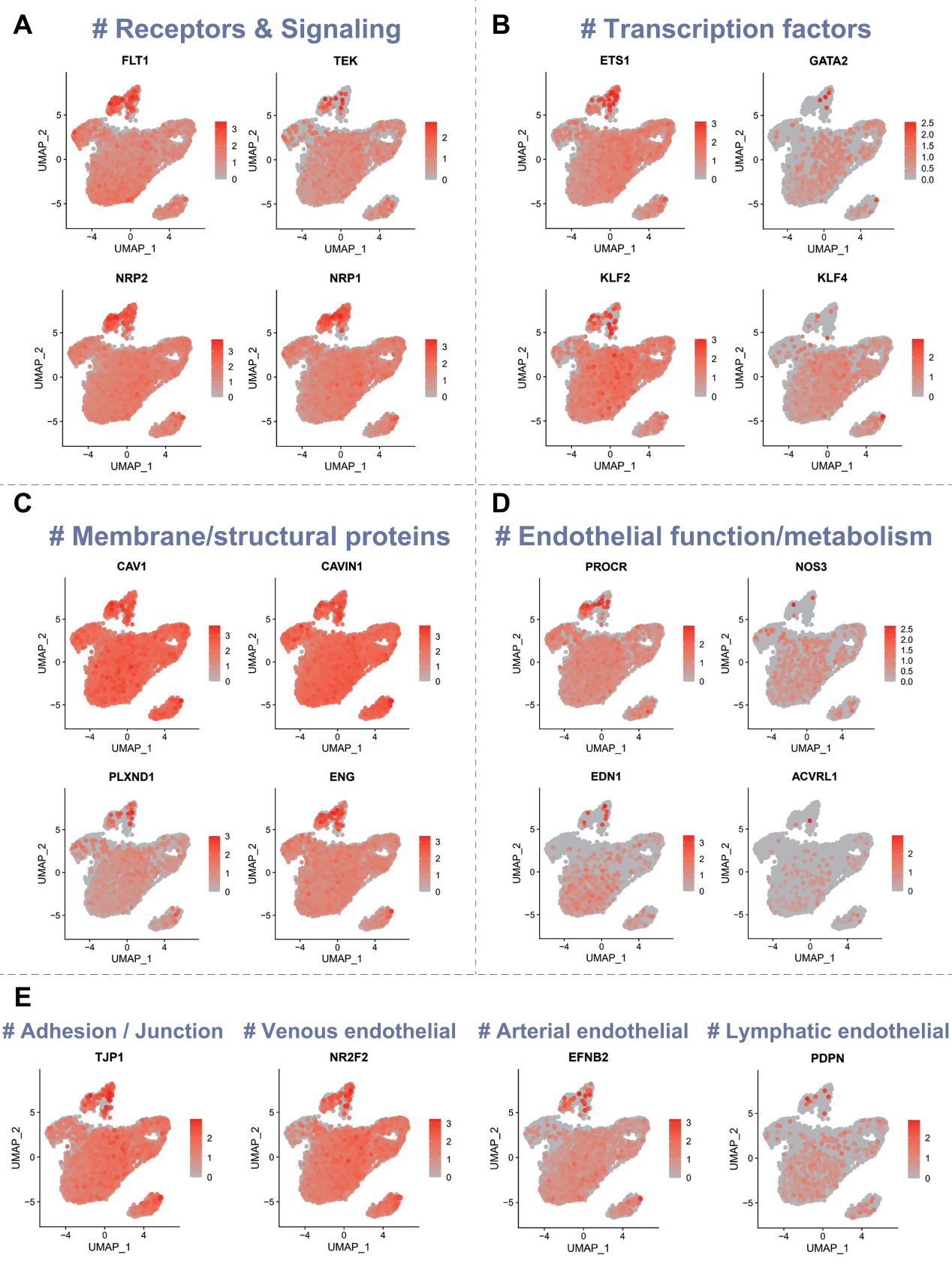


**Supplementary Figure 8** (A) Key receptors and signaling molecules (FLT1/VEGFR1, TEK/TIE2, NRP1/2). (B) Endothelial transcription factors (ETS1, GATA2, KLF2, KLF4). (C) Membrane and junctional components (CAV1, CAVIN1, PLXND1, ENG, TJP1). (D) Genes involved in endothelial function and metabolism (PROCR, NOS3/eNOS, EDN1, ACVRL1). (E) Venous/arterial subtype signals (strong NR2F2, moderate EFNB2; low lymphatic PDPN).

**
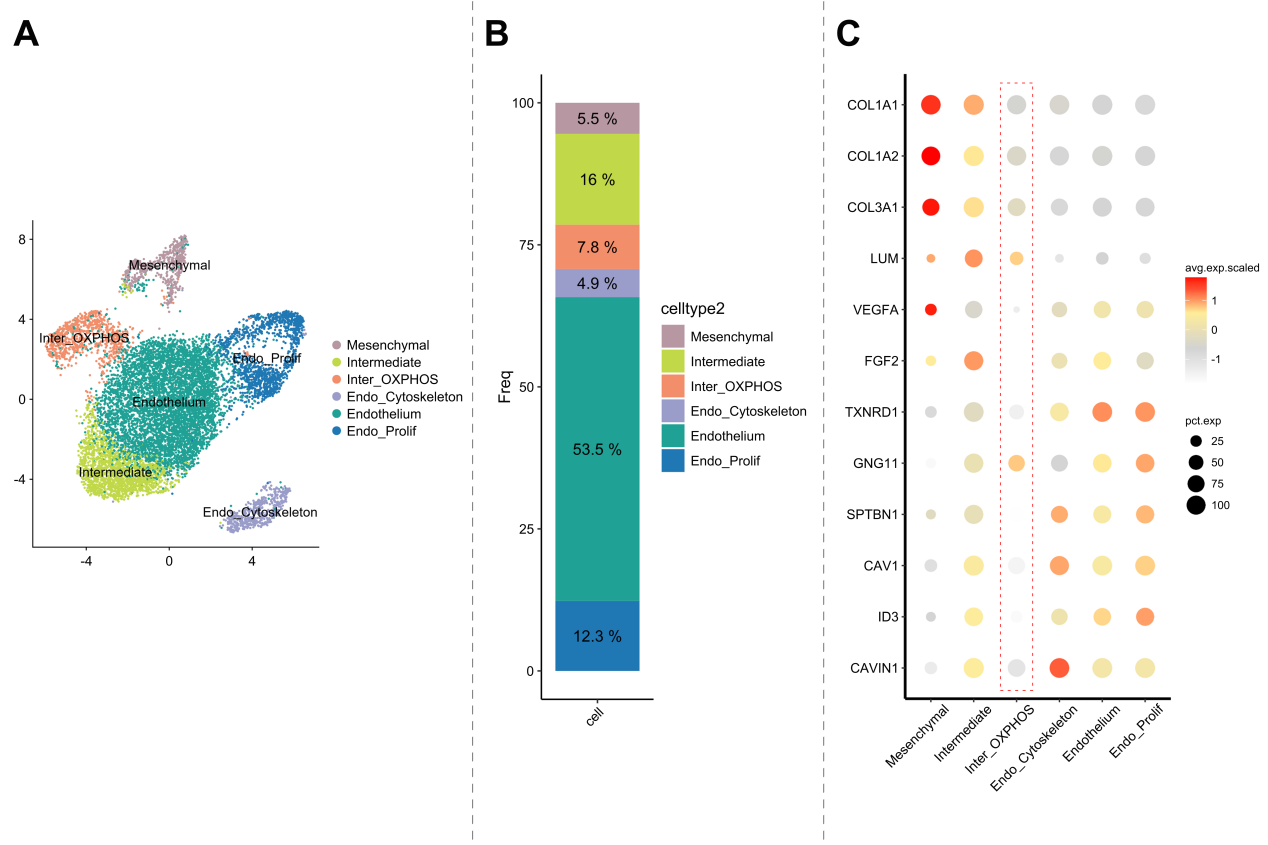
**

**Supplementary Figure 9** (A-B) immunophenotyping revealed that >70% of the cells underwent conversion to the endothelial lineage. (C) Cluster 3 before pseudotemporal ordering.
